# Tripterygium Glycosides Tablet Ameliorates Renal Tubulointerstitial Fibrosis via the Toll-Like Receptor 4/Nuclear Factor Kappa B Signaling Pathway in High-Fat Diet Fed and Streptozotocin-Induced Diabetic Rats

**DOI:** 10.1155/2015/390428

**Published:** 2015-08-12

**Authors:** Ze-jun Ma, Xiao-na Zhang, Li Li, Wei Yang, Shan-shan Wang, Xin Guo, Pei Sun, Li-ming Chen

**Affiliations:** 2011 Collaborative Innovation Center of Tianjin for Medical Epigenetics, Key Laboratory of Hormone and Development (Ministry of Health), Metabolic Disease Hospital & Tianjin Institute of Endocrinology, Tianjin Medical University, Tianjin 300070, China

## Abstract

Tripterygium glycosides tablet (TGT) is a Chinese traditional medicine that has been shown to protect podocytes from injury and reduce the proteinuria. The aim of this study was to assess the effect of TGT on renal tubulointerstitial fibrosis and its potential mechanism in high-fat diet fed and STZ-induced diabetic rats. Rats were randomly divided into normal control rats (NC group), diabetic rats without drug treatment (DM group), and diabetic rats treated with TGT (1, 3, or 6 mg/kg/day, respectively) for 8 weeks. The results showed that 24 h proteinuria and urinary N-acetyl-glucosaminidase (NAG) in diabetic rats were decreased by TGT treatment without affecting blood glucose. Masson's trichrome stains showed that apparent renal tubulointerstitial fibrosis was found in DM group, which was ameliorated by TGT treatment. The expression of *α*-SMA was significantly decreased, accompanied by increased expression of E-cadherin in TGT-treated rats, but not in untreated DM rats. Further studies showed that TGT administration markedly reduced expression of TLR4, NF-*κ*B, IL-1*β*, and MCP-1 in TGT-treated diabetic rats. These results showed that TGT could ameliorate renal tubulointerstitial fibrosis, the mechanism which may be at least partly associated with the amelioration of EMT through suppression of the TLR4/NF-*κ*B pathway.

## 1. Introduction

Diabetic nephropathy (DN) is the leading cause of end-stage renal disease (ESRD) and affects approximately 15–25% of T1DM patients and 30–40% of T2DM patients [1–3]. DN is characterized by hypertension, persistent proteinuria, progressive loss of renal function, and an increased risk of cardiovascular morbidity and mortality. Although glomerulosclerosis is a cardinal feature of DN, accumulating evidence indicates that tubulointerstitial injury is a better predictor of progression of renal lesion compared with the glomerular injury [4]. Renal tubulointerstitial fibrosis is one of the major pathological characteristics of DN, and it is also the common pathway in progressive renal disease leading to loss of renal function and ESRD eventually. Current available therapeutic interventions, including intensive control of blood glucose, optimal control of blood pressure, interruption of the renin-angiotensin-aldosterone system through the use of angiotensin converting enzyme inhibitors (ACEI), and/or angiotensin II type 1 receptor blockers (ARBs), along with cholesterol lowering agents and dietary modification, are unable to effectively reverse or even delay the progression of DN in a substantial proportion of patients [5–7]. This indicates that, in addition to the above mentioned factors, other pathways are involved in the pathogenesis of DN, and more effective therapies to prevent the progression of DN need to be explored.

DN was traditionally considered as a noninflammatory disease; however, accumulating evidences from experimental and clinical studies have shown that immunologic and inflammatory mechanisms play a pivotal role in promoting the development and progression of DN [8–10]. Toll-like receptors (TLRs) are a conserved family of pattern recognition receptors that play an important role in innate immunity and inflammation [11]. At least 10 TLRs currently have been identified in humans [12]. TLR4, a member of the TLRs, has increasingly been shown to play an important role in the activation of NF-*κ*B. The activation of NF-*κ*B has been shown to enhance the expression of proinflammatory cytokines, cell adhesion molecule proteins, and chemokines, which in turn initiate local inflammation and leukocyte accumulation. It is well known that these proinflammatory cytokines and chemokines are associated with the pathogenesis of DN in patients [13, 14]. Furthermore, recent evidences have shown that TLR4-mediated pathway can promote tubulointerstitial inflammation in DN, and TLR4 antagonist may slow the progression of DN by attenuating renal tubulointerstitial inflammation [15–18]. These studies strongly indicate that TLR4-mediated inflammatory processes represent a novel mechanism underlying the pathogenesis of DN. Therefore, it may be a novel and effective therapeutic strategy to reduce renal inflammation and ameliorate DN by suppressing the TLR4/NF-*κ*B pathway [19].

Tripterygium glycosides tablet (TGT) is a traditional Chinese medicine, isolated from the plant* Tripterygium wilfordii* Hook F that has been used for many years in the treatment of chronic kidney disease due to its immunosuppressive and anti-inflammatory effects [20, 21]. Our recent studies have also demonstrated that TGT can alleviate the local inflammation in kidney and reduce the albuminuria of rats with diabetic nephropathy [22]. However, nearly all of the previous researches on TGT's nephroprotective effect mainly focused on glomerulus structure and function, while the effect and possible mechanisms of TGT on renal tubulointerstitial injury in DN have not been fully established. In the present study, we utilized a rat model of type 2 diabetes mellitus induced by HFD/STZ and determined whether TGT could suppress the activation of TLR4/NF-*κ*B pathways and thereby attenuate renal tubulointerstitial injury.

## 2. Materials and Methods

### 2.1. Drugs and Reagents

TGT was purchased from Chinese National Institute for the Control of Pharmaceutical and Biological Products. Streptozotocin (STZ) was purchased from Sigma Chemical Company (St. Louis, MO, USA). TLR4, nuclear factor kappa B (NF-*κ*B)-p-p-65, IL-1*β*, MCP-1, E-cadherin, and *α*-smooth muscle actin (*α*-SMA) antibodies were purchased from Cell Signaling Company or Santa Cruz Biotechnology. *β*-actin as loading control was purchased from Thermo pierce. Blood glucose test machine and strips were purchased from Changsha Sinocare Inc. (Chang-sha, China). Immunohistochemical detection kit and SYBRGreen PCR kit were from Zhongshan Company (Beijing, China), Trizol total RNA extraction kit was from Thermo Scientific, USA, and ABI-7300 real-time detection instrument was from Applied Biosystems (CA, USA).

### 2.2. Animals

Male Sprague-Dawley (SD) rats (*n* = 50, 6-week-old) weighing 170 ± 10 g were purchased from Beijing HFK Bio-Technology Co. Ltd. All animals were housed in a 12 h light/dark altered room at a controlled temperature (23°C ± 2°C) and relative humidity (50–60%). The study was approved by the ethical committee of Tianjin Medical University, and all procedures involving rats were conducted according to the Guide for the Care and Use of Laboratory Animals of the National Institutes of Health as well as the guidelines of the Animal Welfare Act. Food and water were available ad libitum.

### 2.3. Animal Model and Treatment Protocols

T2DM was induced in rats with high-fat diet (HFD) followed by a single tail intravenous injection of STZ as previously reported [23]. All rats were first fed with regular rodent chow for 1 week to adapt to the environment. The animals were then randomly assigned to normal control group (NC group, *n* = 10), which were fed with standard diet, and HFD group (*n* = 40), which were allowed free access to the high-fat diet to induce dyslipidemia for 6 weeks. The high-fat diet consisted of 78.7% standard diet, 10% glucose, 10% animal fat, 1% TC, and 0.3% sodium cholate. Then, the HFD rats were administered a single tail intravenous injection of 30 mg/kg STZ dissolved in 0.1 M citrate-phosphate buffer (pH 4.5) after overnight fasting. Normal control rats were injected with citrate-phosphate buffer alone. About 72 h after STZ injection, the diabetic model was considered to be successful when the random blood glucose was >16.7 mmol/L for three consecutive tests. The diabetic rats were then randomly divided into four groups: diabetic rats without drug treatment (DM group, *n* = 10) and diabetic rats treated with TGT at a low-dose group (TGT of 1 mg/kg, *n* = 10), medium-dose group (TGT of 3 mg/kg, *n* = 10), and high-dose group (TGT of 6 mg/kg, *n* = 10), respectively. TGT dissolved in 0.5% dimethyl sulfoxide (DMSO) was diluted to the appropriate concentrations with saline and was given by daily gastric gavage for 8 weeks. The rats in NC group and DM group received equal volumes of saline (also contained 0.5% DMSO) every day. All rats were allowed free access to food and water during the experiment.

At the end of study, all rats were placed in individual metabolic cage to collect 24 h urine samples for the measurement of the urinary protein and urine NAG (N-acetyl-*β*-d-glucosaminidase). Urinary protein was determined by the Bradford method and urine NAG was determined by an assay kit (Jiancheng, Nanjing, China). The overnight-fasted rats were weighed and then anesthetized with intraperitoneal injection of sodium pentobarbital (30 mg/kg body weight). Blood samples were obtained from the retroorbital venous plexus at sacrifice and were centrifuged at 3000 g/min for 15 min. Serum was separated for measuring blood glucose, total triglyceride (TG), total cholesterol (TC), blood urea nitrogen (BUN), serum creatinine (Scr), aspartate transaminase (AST), and alanine transaminase (ALT) by an automatic biochemistry analyzer (CD-1600CS, Abbott Labs, USA). The kidneys were immediately removed, weighed, and frozen in liquid nitrogen and then stored at −80°C or fixed in 10% neutral buffered formalin (NBF).

### 2.4. Masson's Trichrome Staining of Renal Tissues

The renal tissue was fixed in 10% NBF and embedded in paraffin, and 4 *μ*m sections were stained with Masson's trichrome to detect the area of renal interstitial fibrosis. After staining, the sections were evaluated at ×400 magnification by two investigators in a blinded manner using IDA-2000 high-resolution digital microscope and image analysis system (Konghai tec, Beijing, China). Green staining areas were measured and the ratio of total green area over the entire field of vision was assessed to represent the percentage of the area of renal tubulointerstitial fibrosis. For each slide, 10 randomly selected nonoverlapping fields of renal cortex without glomeruli and large vessels were measured and calculated, and the average of each group was analyzed.

### 2.5. Immunohistochemistry Staining of Renal Tissues

Formalin-fixed, paraffin-embedded sections (4 *μ*m thick) were deparaffinized by xylene and hydrated by graded ethanol. Heat-induced antigen retrieval was conducted at 95°C by microwave in 10 mmol/L sodium citrate buffer (pH 6.0) for 5 min. After that, the sections were blocked with 3% H_2_O_2_ for 15 min and incubated overnight at 4°C with the following antibodies diluted with phosphate-buffered saline (PBS): anti-TLR4 (1 : 100), anti-NF-*κ*B (1 : 100), anti-IL-1*β* (1 : 200), anti-MCP-1 (1 : 100), anti-E-cadherin (1 : 100), and anti-*α*-SMA (1 : 100). Then, the sections were washed three times in PBS and incubated for 45 min with the secondary antibody; subsequently, the sections were visualized with a diaminobenzidine (DAB) kit and counterstained with haematoxylin. The stained sections were examined in a blinded manner using light microscopy (Olympus BX-50, Olympus Optical, Tokyo, Japan) in ten randomly selected cortical sections at a magnification ×400. The semiquantitative analysis was scored using Image-Pro plus 6.0 image analysis software to calculate the number of positive cells and percentage of positive area of the tubular-interstitial area.

### 2.6. Real-Time Quantitative PCR

Total RNA was extracted from kidney tissues using TRIzol reagent (Invitrogen, USA) according to the manufacturer's instructions. Template complementary DNA (cDNA) was synthesized using a reverse transcription system kit (Takara, Dalian, China). The PCR reactions were performed on the CFX96 real-time PCR system (Bio-Rad, USA) with a SYBR Green PCR reagent kit (SYBR PremixEx TaqTM II, Takara, Japan). The primers used in this study were obtained from Genscript Corp (Nanjing, China), and their sequences were as follows: for TLR4 5′-CCGCTCTGGCATCATCTTCA-3′ and 5′-TCCCACTCGAGGTAGGTGTT-3′; for NF-*κ*B 5′-TGTCAACATTAGCGAGGGT-3′ and 5′-CCTCGTTTGCACTGTTATG-3′; for IL-1*β* 5′-GAGTTCCGTTTCTACCTG-3′ and 5′-AGGAGAGCATTGGAAGTTG-3′; for MCP-1 5′-CCTCCACCACTATGCAGGTC-3′ and 5′-CAGCCGACTCATTGGGATCA-3′; for GAPDH 5′-GCAAGTTCAACGGCACAG-3′ and 5′-GCCAGTAGACTCCACGACAT-3′. The threshold cycles (Ct) for the target gene and the endogenous control were measured for each sample. The relative mRNA expression was carried out using the 2^−ΔΔCT^ method and normalized to controls.

### 2.7. Western Blotting

The total protein of the kidney tissues was extracted with a Pro-Prep Protein Extraction Solution (Intron Biotechnology, Gyeonggi-Do, Korea) following the manufacturer's instructions. The protein concentration was measured using a BCA protein assay (Pierce Biotechnology, Rockford, IL, USA). Equal amounts of protein sample (20 *μ*g) were separated by electrophoresis on 12% SDS-PAGE gels and then transferred to PVDF membranes. After being blocked in TBS containing 5% nonfat milk for 2 h at room temperature, the membranes were incubated overnight at 4°C with the primary antibodies against the following proteins: TLR4 (diluted 1 : 1.000); NF-*κ*B p65 (1 : 1.000); IL-1*β* (1 : 2.000); MCP-1 (diluted 1 : 1.000); *α*-SMA (1 : 2.000); E-cadherin (1 : 1.000); and *β*-actin (1 : 8.000). After the blots were washed with TBST, they were incubated with a secondary antibody. The protein bands were visualized by using ECL kit (Bio-Rad, Hercules, CA) and then captured on X-ray film. Housekeeping protein *β*-actin was used as a loading control. The density of each band was quantified using Quantity One software (Bio-Rad Laboratory, Hercules, CA) and normalized to their respective control.

### 2.8. Statistical Analysis

SPSS 16.0 software was used for statistical analysis. All the values were expressed as mean ± SD. Data were analyzed using one-way analysis of variance (ANOVA) or the LSD *t*-test. *p* < 0.05 was considered statistically significant.

## 3. Results

### 3.1. Effects of TGT Treatment on Blood Biochemical Parameters

The blood biochemical parameters were presented in Table 1. During the study period, the levels of blood glucose, TC, and TG were significantly higher in both DM and TGT-treated rats compared with the NC rats (*p* < 0.05). However, there were no significant differences between DM and TGT-treated groups (*p* > 0.05), indicating that TGT had no effects on blood glucose and blood lipid. In addition, the AST and ALT levels did not differ among the five groups (*p* > 0.05). Furthermore, Scr and BUN did not change in these groups by the end of the experiment (*p* > 0.05).

### 3.2. Effect of TGT on Urinary Protein, NAG, and KW/BW

The 24 h urine protein was significantly increased in the DM rats compared with the NC rats (*p* < 0.05), which was attenuated in a dose-dependent manner after TGT administration for 8 weeks (*p* < 0.05). NAG, one of the urinary tubular injury biomarkers, was significantly increased in the DM rats compared with the NC rats but was markedly reduced by TGT treatment (*p* < 0.05). The kidney weight-to-body weight ratio (KW/BW) was significantly higher in the DM rats compared with the NC rats (*p* < 0.05); by contrast, KW/BW was significantly reduced in the TGT treatment group (*p* < 0.05) but still remained significantly higher in comparison with normal control (Table 1).

### 3.3. Effects of TGT on Renal Tubulointerstitial Fibrosis

**Figure 1:**
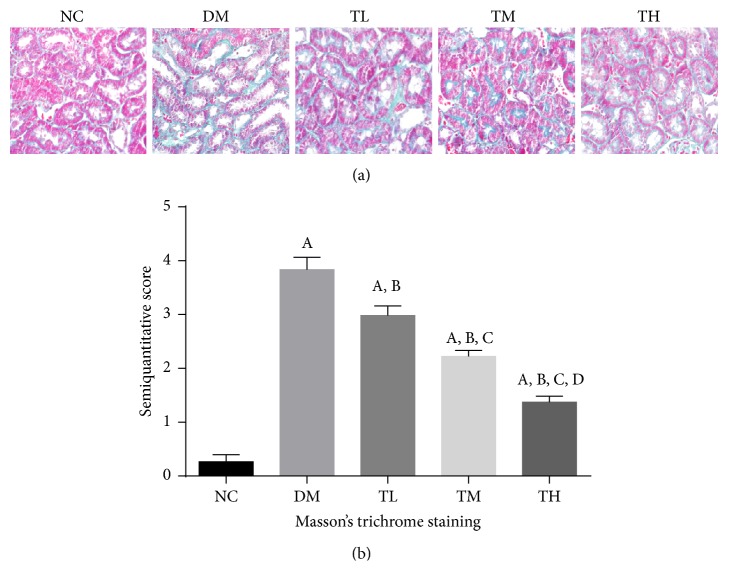
Pathomorphology of renal tissue in rats to evaluate tubulointerstitial fibrosis (Masson ×400). (a) Representative Masson's trichrome stained sections. (b) Semiquantitative score of tubulointerstitial fibrosis for different groups. NC: normal control; DM: diabetic rats without drug treatment; TL: low-dose TGT (1 mg/kg/day); TM: medium-dose TGT (3 mg/kg/day); TH: high-dose TGT (6 mg/kg/day). ^A^ *p* < 0.05 versus NC group, ^B^ *p* < 0.05 versus DM group, ^C^ *p* < 0.05 versus TL group, and ^D^ *p* < 0.05 versus TM group.

Fibrosis of the tubulointerstitial area was examined by Masson's trichrome stains (Figure 1). For rats in the NC group, there was a very small amount of the tubulointerstitial area stained green. However, for rats in the DM group, green staining in renal interstitial area was significantly larger than that in NC group (*p* < 0.05). In contrast, treatment with TGT for 8 weeks significantly reduced green staining in renal interstitial area in a dose-dependent matter (*p* < 0.05). These results suggested that TGT could inhibit the renal tubulointerstitial fibrosis in DN.

### 3.4. Effect of TGT on the Renal Expression of E-Cadherin and *α*-SMA

**Figure 2:**
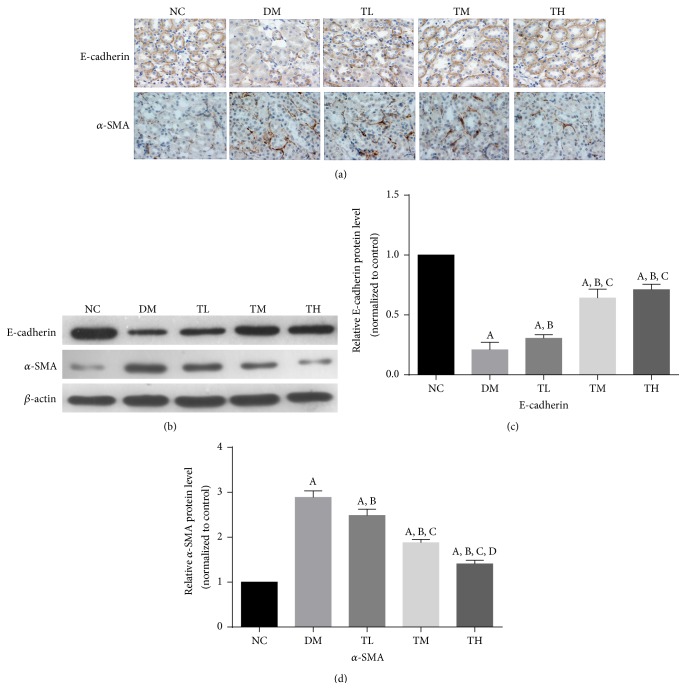
Effect of TGT on E-cadherin and *α*-SMA localization and expression in diabetic rat kidney by immunohistochemistry and western blot. (a) Immunostaining of E-cadherin and *α*-SMA was shown for different groups. (b) Representative western blotting analysis of E-cadherin and *α*-SMA was shown for different groups. (c-d) Densitometric results of E-cadherin and *α*-SMA, as determined by western blot. NC: normal control; DM: diabetic rats without drug treatment; TL: low-dose TGT (1 mg/kg/day); TM: medium-dose TGT (3 mg/kg/day); TH: high-dose TGT (6 mg/kg/day). ^A^ *p* < 0.05 versus NC group, ^B^ *p* < 0.05 versus DM group, ^C^ *p* < 0.05 versus TL group, and ^D^ *p* < 0.05 versus TM group.

Next, we examined the effect of TGT therapy on the expression of E-cadherin and *α*-SMA (Table 2, Figure 2). Immunohistochemistry results showed that there was only very week staining of *α*-SMA in renal tubular epithelial cells in NC group. However, those were significantly increased in the tubulointerstitium of diabetic rats in comparison with the NC rats; TGT administration at the dose of 1, 3, or 6 mg·kg^−1^·d^−1^ could inhibit the expression of *α*-SMA in the kidney of diabetic rats. Compared with the normal group, the number of E-cadherin positive epithelial cells was obviously decreased in the DM groups, and it was partly recovered with the TGT administration.

Similarly, western blot analysis showed that the expression of *α*-SMA was significantly increased in DM group compared with NC group (*p* < 0.05). However, treatment with TGT decreased *α*-SMA protein expression in a dose-dependent manner (*p* < 0.05). Compared with the normal group, the expression of the E-cadherin was obviously decreased in the DM groups (*p* < 0.05), and it was significantly increased with the TGT administration (*p* < 0.05).

### 3.5. Effects of TGT on Inflammation in the Kidney of Diabetic Rats

**Figure 3:**
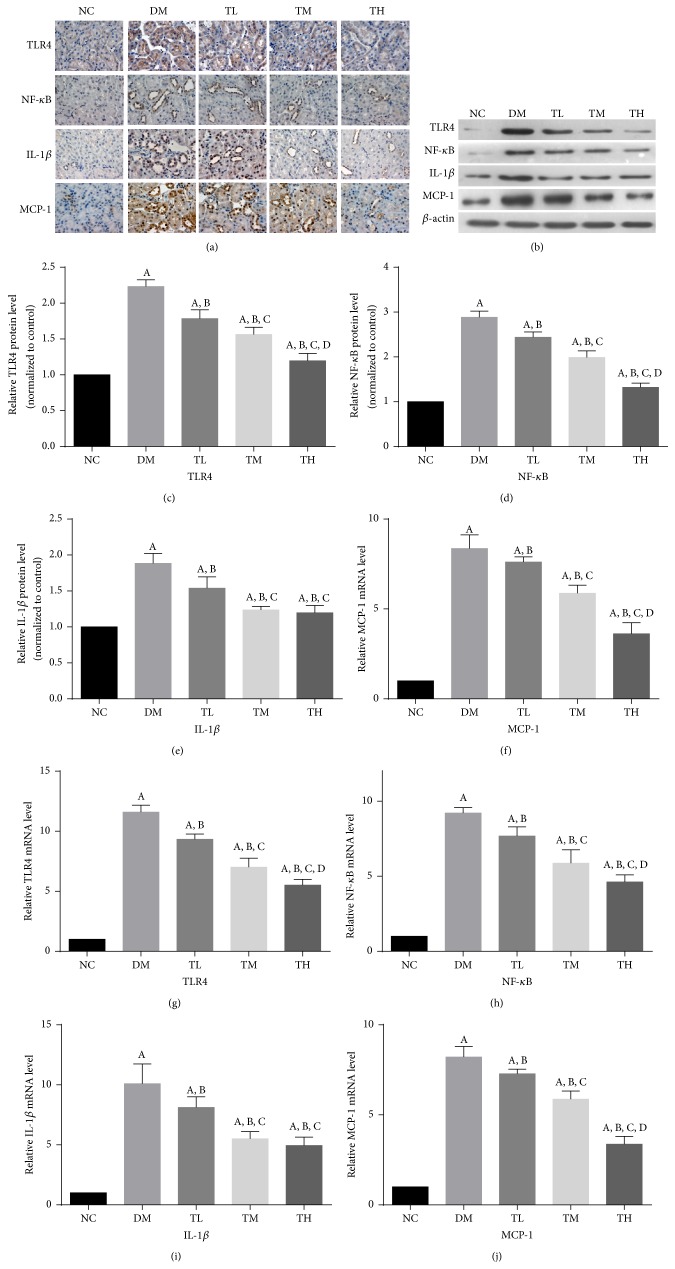
TGT attenuated diabetes-induced renal inflammation. (a) Immunostaining of TLR4, NF-*κ*B, IL-1*β*, and MCP-1 was shown for different groups. (b) Representative western blotting analysis of TLR4, NF-*κ*B, IL-1*β*, and MCP-1 was shown for different groups. (c–f) Densitometric results of TLR4, NF-*κ*B, IL-1*β*, and MCP-1, as determined by western blot. (g–j) The mRNA expressions of TLR4, NF-*κ*B, IL-1*β*, and MCP-1 were shown for different groups. NC: normal control; DM: diabetic rats without drug treatment; TL: low-dose TGT (1 mg/kg/day); TM: medium-dose TGT (3 mg/kg/day); TH: high-dose TGT (6 mg/kg/day). ^A^ *p* < 0.05 versus NC group, ^B^ *p* < 0.05 versus DM group, ^C^ *p* < 0.05 versus TL group, and ^D^ *p* < 0.05 versus TM group.

To investigate whether the renoprotection of TGT results from its anti-inflammatory effect, we performed immunostaining, real-time PCR, and western blotting to investigate expression of TLR4 as well as NF-*κ*b, IL-1*β*, and MCP-1 in the kidneys (Table 2, Figure 3).

Nearly no TLR4 staining was seen in the renal tubular epithelial cells of normal rats on immunohistochemistry but was highly expressed in the renal tubular epithelial cells of diabetic rats. TLR4 staining was markedly reduced in the TGT-treated diabetic rats dose-dependently. Likewise, the expression of NF-*κ*b, IL-1*β*, and MCP-1 was dramatically increased in the tubulointerstitial areas of diabetic rats compared with the normal control rats. Consistently, treatments with TGT reduced NF-*κ*b, IL-1*β*, and MCP-1 expression in the tubulointerstitium.

The expressions of TLR4, NF-*κ*B, IL-1*β* and MCP-1 in kidney were all significantly increased in DM rats compared with the NC rats (*p* < 0.05). However, the expressions of TLR4, NF-*κ*B, IL-1*β*, and MCP-1 in kidney were decreased in TGT-treated diabetic rats compared with rats in diabetic group (*p* < 0.05).

## 4. Discussion

DN, one of the microvascular complications of diabetes, is characterized by a series of renal structure changes including basement membrane thickening, mesangial expansion, glomerulosclerosis, and tubulointerstitial fibrosis [24]. TGT is a Chinese traditional medicine that has long been used to treat chronic kidney diseases including DN, due to its immunosuppressive and anti-inflammatory effects [20, 21]. We have previously reported that TGT decreased albuminuria in type 2 diabetic patients. Recently we demonstrated that TGT ameliorated diabetic nephropathy, decreased albuminuria, and alleviated glomerular injuries without changing blood glucose levels in type 2 diabetic rats [22], which suggest that the anti-inflammatory efficacy of TGT might be beneficial for the treatment of DN. However, TGT's anti-inflammatory effect on renal tubulointerstitial fibrosis has been less reported.

The present study was undertaken to examine whether TGT can ameliorate renal tubulointerstitial fibrosis and to investigate the potential mechanisms. In this study, we found that diabetic rats showed renal tubulointerstitial fibrosis in histomorphological and biochemical aspects, and administration of TGT dose-dependently attenuated renal tubulointerstitial fibrosis and decreased proteinuria. In addition, the beneficial effect of TGT on renal tubulointerstitial fibrosis might be related, at least in part, to the inhibition of TLR4/NF-*κ*B signaling and downregulation of expression of inflammatory mediator without affecting blood glucose and blood lipid levels of diabetic rats. These results demonstrated that TGT is an effective agent for ameliorating diabetic renal tubulointerstitial fibrosis through its anti-inflammatory effects.

Although the glomerulus, particularly the mesangium, has been the focus of intense investigation in diabetes, renal tubulointerstitial fibrosis is also a major feature of diabetic nephropathy and an important predictor of renal dysfunction [25]. The histopathology of tubulointerstitial fibrosis includes renal tubular epithelial cell loss, myofibroblast accumulation, inflammatory cell infiltration, and extracellular matrix (ECM) deposition [26, 27]. Epithelial-mesenchymal transition (EMT) has long been considered to be an important pathway in myofibroblast production and is a key mechanism in the pathogenesis and progression of renal interstitial fibrosis [28, 29]. In EMT, the loss of tubular epithelial cell adhesion molecules, such as E-cadherin, is replaced by the *α*-SMA, which is one of the interstitial myofibroblast activation markers [30]. Myofibroblast is the principal cell responsible for the deposition of ECM and fibrosis under pathological conditions. In the present study, we found that the expression of *α*-SMA was significantly decreased, accompanied by increased expression of E-cadherin in TGT-treated DM rats in a dose-dependent matter, but not in untreated DM rats, suggesting that the inhibition of EMT could be an important mechanism by which TGT exerts its effectiveness on renal tubulointerstitial fibrosis.

There is increasing clinical and experimental evidence showing that inflammatory and immunologic processes play an important role in initiating and extending diabetic tubulointerstitial fibrosis [16, 31, 32]. Infiltration of inflammatory cell and accumulation of macrophages stimulated by innate immune response are found to be crucial during the development of tubulointerstitial fibrosis [33]. TLRs are one of the receptor types of the innate immune system, and they play an important role in the regulation and linking of innate and acquired immunity, as well as in the development of inflammatory and immune responses [34]. TLR4, a member of the TLRs, might be a molecular link between chronic inflammation and metabolic syndrome associated disorders such as hyperglycemia, dyslipidemia, and hemodynamic abnormalities, which have been implicated to be the risk factors contributing to DN [15, 35]. Renal tubular epithelial cells are nonimmune cells and express TLR4 in vitro [36]. Tsuboi et al. [37] first reported the expression of TLR4 in primary cultures of mouse cortical renal epithelial cells. Other studies have confirmed that TLR4 are expressed in renal tubule cells from mouse, rat, and human kidneys [38–40]. Recent evidences have shown that TLR4-mediated signaling pathway can mediate monocyte/macrophage recruitment and tubulointerstitial damage induced by the immune response in DN [17]. After activation, TLR4 can activate the NF-*κ*B signaling pathway, which promotes the protein expression and activity of its downstream inflammatory cytokines, such as transforming growth factor *β*1 (TGF-*β*1), tumor necrosis factor-*α* (TNF-*α*), and IL-1*β* in the kidney of diabetic rats [41]. These cytokines can further promote fibroblast proliferation, *α*-SMA expression, and transdifferentiation into myofibroblasts that synthesize and secrete collagen, which results in extracellular matrix deposition and tubulointerstitial fibrosis.

In the present study, we found that diabetic rats presented remarkable renal tubulointerstitial fibrosis accompanied by an increase of TLR4, NF-*κ*B IL-1*β*, and MCP-1 expression at both gene and protein levels in comparison with the normal rats. In addition, we found that the expression of TLR4, NF-*κ*B IL-1*β*, and MCP-1 was located in renal tubular epithelial cells as shown by immunohistochemistry, which strongly supported a role for TLR4 involved in occurrence and development of diabetic renal tubulointerstitial fibrosis, which is consistent with the previous study. Furthermore, in this study, we demonstrated that TGT treatment obviously decreased the renal expression of TLR4 with corresponding decrease in NF-*κ*B activation and inflammatory cytokine production in a dose-dependent manner compared with rats in the diabetic group. These results indicated that TGT could prevent the progression of renal tubulointerstitial fibrosis, and downregulation of TLR4, NF-*κ*B IL-1*β*, and MCP-1 may be one of the important reasons. TGT may represent a novel strategy to inhibit TLR4-mediated inflammatory processes in renal tubulointerstitial fibrosis. However, these novel findings need to be confirmed in patients and in vitro experiment.

In this study, we found that the TGT at the doses of 1, 3, and 6 mg/kg/day were well tolerated by the diabetic rats compared to control, which is consistent with the results of Ma et al. [42]. However, the potential adverse effects on the liver with TGT treatment were reported in clinical studies [20, 21]. This liver injury has been reported to present as increased serum ALT or AST level, be associated with the dosage of TGT, and be resolved by reduction or withdrawal of the agent.

In summary, our research demonstrated that TGT treatment could ameliorate renal tubulointerstitium fibrosis in diabetic rats, the mechanism of which may be at least partly associated with the amelioration of EMT through suppression of the TLR4/NF-*κ*B pathway.

## Figures and Tables

**Table 1:** Physical and biochemical parameters of the rats in different groups (mean ± SD).

	NC (*n* = 10)	DM (*n* = 10)	TL (*n* = 10)	TM (*n* = 10)	TH (*n* = 10)
Blood glucose (mmol/L)	6.0 ± 0.2	28.2 ± 3.0^a^	25.8 ± 6.9^a^	27.6 ± 5.7^a^	29.0 ± 3.1^a^
TC (mmol/L)	3.7 ± 1.2	6.5 ± 1.9^a^	6.6 ± 2.1^a^	6.4 ± 2.2^a^	6.2 ± 2.0^a^
TG (mmol/L)	1.5 ± 1.2	4.1 ± 1.8^a^	4.0 ± 2.2^a^	3.7 ± 1.8^a^	3.8 ± 1.4^a^
ALT (mmol/L)	49.7 ± 7.5	55.1 ± 5.1	53.8 ± 10.9	54.9 ± 9.7	54.2 ± 8.9
AST (mmol/L)	75.1 ± 7.1	79.4 ± 6.4	76.8 ± 7.5	79.5 ± 5.9	82.2 ± 6.3
BUN (mmol/L)	6.9 ± 2.0	6.7 ± 1.6	6.9 ± 1.3	7.0 ± 1.9	6.8 ± 1.2
Scr (mmol/L)	62.8 ± 8.4	61.3 ± 7.3	62.8 ± 9.5	61.1 ± 8.1	61.5 ± 7.8
24-h urine protein (mg/24 h)	45.7 ± 14.5	253.7 ± 33.0^a^	183.5 ± 28.7^a,b^	123.8 ± 24.8^a,b,c^	119.0 ± 22.0^a,b,c^
NAG	12.3 ± 4.1	59.5 ± 9.3^a^	41.7 ± 8.9^a,b^	32.9 ± 7.5^a,b,c^	29.4 ± 7.2^a,b,c^
KW/BW	3.21 ± 0.18	6.78 ± 0.48^a^	5.90 ± 0.55^a,b^	4.89 ± 0.3^a,b,c^	4.55 ± 0.38^a,b,c^

TC: total cholesterol; TG: total triglycerides; ALT: alanine transaminase; AST: aspartate transaminase; BUN: blood urea nitrogen; Scr: serum creatinine; NC: normal control; DM: diabetic rats without drug treatment; TL: low-dose TGT (1 mg/kg/day); TM: medium-dose TGT (3 mg/kg/day); TH: high-dose TGT (6 mg/kg/day). ^a^ *p* < 0.05 versus NC group, ^b^ *p* < 0.05 versus DM group, and ^c^ *p* < 0.05 versus TL group.

**Table 2:** Expression of E-cadherin, *α*-SMA, TLR4, NF-*κ*B p65, IL-1*β*, and MCP-1 in the tubulointerstitial tissues by immunohistochemistry (mean ± SD).

	NC (*n* = 10)	DM (*n* = 10)	TL (*n* = 10)	TM (*n* = 10)	TH (*n* = 10)
E-cadherin	3.21 ± 0.58	0.27 ± 0.16^a^	1.01 ± 0.29^a,b^	2.46 ± 0.37^a,b,c^	2.62 ± 0.41^a,b,c^
*α*-SMA	0.24 ± 0.15	3.19 ± 0.29^a^	2.63 ± 0.21^a,b^	2.14 ± 0.18^a,b,c^	1.57 ± 0.20^a,b,c,d^
TLR4	0.19 ± 0.12	2.92 ± 0.17^a^	2.24 ± 0.18^a,b^	1.73 ± 0.15^a,b,c^	1.29 ± 0.14^a,b,c,d^
NF-*κ*B	0.35 ± 0.19	4.12 ± 0.21^a^	3.81 ± 0.22^a,b^	3.09 ± 0.17^a,b,c^	1.94 ± 0.19^a,b,c,d^
IL-1*β*	0.31 ± 0.11	3.48 ± 0.24^a^	2.89 ± 0.15^a,b^	2.19 ± 0.15^a,b,c^	1.96 ± 0.17^a,b,c^
MCP-1	0.36 ± 0.09	3.07 ± 0.26^a^	2.31 ± 0.12^a,b^	1.54 ± 0.18^a,b,c^	1.15 ± 0.15^a,b,c,d^

NC: normal control; DM: diabetic rats without drug treatment; TL: low-dose TGT (1 mg/kg/day); TM: medium-dose TGT (3 mg/kg/day); TH: high-dose TGT (6 mg/kg/day). ^a^ *p* < 0.05 versus NC group, ^b^ *p* < 0.05 versus DM group, ^c^ *p* < 0.05 versus TL group, and ^d^ *p* < 0.05 versus TM group.
